# ICSI outcomes for infertile men with severe or complete asthenozoospermia

**DOI:** 10.1186/s12610-022-00155-x

**Published:** 2022-04-05

**Authors:** Tong Chen, Demin Fan, Xianlong Wang, Changlin Mao, Yaru Chu, Haobo Zhang, Wen Liu, Sentai Ding, Qingyong Liu, Mingzhen Yuan, Jiaju Lu

**Affiliations:** 1 Department of Urology, Cheeloo College of Medicine, Shandong Provincial Hospital, Shandong University, 324 Jingwu Road, 250021 Jinan, Shandong P. R. China; 2 Center for Reproductive Medicine, Cheeloo College of Medicine, Shandong University, 250000 Jinan, Shandong P.R. China; 3 Department of Urology, The First Affiliated Hospital of Nanjing Medical University, 210000 Nanjing, P.R. China; 4 Department of Urology, Cheeloo College of Medicine, Shandong Qianfoshan Hospital, Shandong University, 250000 Jinan, Shandong P.R. China; 5 Department of Urology, The First Affiliated Hospital of Nanjing Medical University, 300 Guangzhou Road, 210029 Nanjing, P.R. China

**Keywords:** Asthenozoospermia, Ejaculated spermatozoa, Intracytoplasmic sperm injection (ICSI), Male infertility, Testicular spermatozoa

## Abstract

**Background:**

Severe or complete asthenozoospermia is a rare entity that can lead to male infertility. In this study, we explored whether different extents of severe or complete asthenozoospermia could affect intracytoplasmic sperm injection (ICSI) outcomes and compared the ICSI outcomes using testicular spermatozoa with those using ejaculated spermatozoa in couples with complete asthenozoospermia.

**Results:**

Ninety-seven couples with severe or complete asthenozoospermia who underwent ICSI between January 2014 and December 2018 were included. According to the sperm category used in ICSI, patients were categorized into four groups: ejaculated progressive motile sperm group (Ep-group), ejaculated non-progressive motile sperm group (En-group), ejaculated immotile sperm group (Ei-group), and testicular sperm group (TESE-group). We compared the baseline characteristics, hormone profile, semen parameters, normal fertilization, good-quality embryos on day 3, transferred embryos, and ICSI outcomes in the four groups. The clinical pregnancy rate was significantly increased in the Ep-group (65.4%, *P* = 0.019) and TESE-group (63.6%, *P* = 0.035) compared with that in the Ei-group (23.1%). The ongoing pregnancy rate in the Ei-group was significantly lower than that in the Ep-group (23.1% vs. 61.5%, *P* = 0.041). Moreover, the biochemical pregnancy rate, ongoing pregnancy rate, and live birth rate were much lower in the Ei-group than in the TESE-group (30.8% vs. 63.6%, 23.1% vs. 40.4% and 23.1% vs. 40.4%, respectively).

**Conclusions:**

In couples with complete asthenozoospermia, testicular spermatozoa should be preferred to ejaculated spermatozoa for obtaining a better ICSI outcome. With the appropriate selection of testicular spermatozoa, the extent of severe or complete asthenozoospermia may not affect the ICSI outcomes. Future studies with a larger sample size are warranted to validate these findings.

## Résumé

**Contexte:**

L’asthénozoospermiesévère ou complète est une entité rare qui peut conduire à l’infertilité masculine. Dans cette étude, nous avons exploré si les différentes étendues de l’asthénozoospermie sévère ou complète pouvaient affecter les résultats de l’injection intracytoplasmique de spermatozoïdes (ICSI), et nous avons comparé les résultats de l’ICSI obtenus avec des spermatozoïdes testiculaires à ceux obtenus avec des spermatozoïdes éjaculés chez les couples atteints d’asthénozoospermie complète.

**Résultats:**

Quatre-vingt-dix-sept couples atteints d’asthénozoospermie sévère ou complète qui ont eu une ICSI entre janvier 2014 et décembre 2018 ont été inclus. Selon la catégorie de spermatozoïdes utilisée dans l’ICSI, les patients ont été classés en quatre groupes : groupe de spermatozoïdes mobiles progressifs éjaculés (groupe Ep), groupe de spermatozoïdes mobiles non progressifs éjaculés (groupe En), groupe de spermatozoïdes immobiles éjaculés (groupe Ei) et groupe de spermatozoïdestesticulaires (groupe TESE). Nous avons comparé les caractéristiques de base, le profil hormonal, les paramètres du sperme, la fécondation normale, les embryons de bonne qualité au jour 3, les embryons transférés, et les résultats de l’ICSI dans les quatre groupes. Le taux de grossesse clinique était significativement augmenté dans le groupe Ep (65,4%, *P* = 0,019) et le groupe TESE (63,6%, *P* = 0,035) par rapport à celui du groupe Ei (23,1%). Le taux de grossesse en cours dans le groupe Ei était significativement inférieur à celui du groupe Ep (23,1% contre 61,5%, *P* = 0,041). De plus, le taux de grossesse biochimique, le taux de grossesse en cours et le taux de naissances vivantes étaient beaucoup plus faibles dans le groupe Ei que dans le groupe TESE (30,8 % vs 63,6%,23,1 % vs 40,4 % et 23,1 % vs 40,4 %, respectivement).

**Conclusions:**

Chez les couples atteints d’asthénozoospermie complète, les spermatozoïdes testiculaires devraient être préférés aux spermatozoïdes éjaculés pour obtenir un meilleur résultat en ICSI. Avec une sélection appropriée des spermatozoïdes testiculaires, l’étendue de l’asthénozoospermie sévère ou complète pourrait ne pas affecter les résultats de l’ICSI. De futures études avec des échantillons de plus grande taille sont donc justifiées pour valider ces résultats.

**Mots-clés:**

Asthénozoospermie; spermatozoïdes éjaculés ; injection intracytoplasmique de spermatozoïdes (ICSI) ; infertilité masculine ; spermatozoïdes testiculaires.

## Introduction

Infertility is a worldwide public health concern, affecting approximately 15% of couples during their childbearing years [1]. Among these couples, up to 50% are related to male-factor infertility [2]. Asthenozoospermia, the leading cause of male infertility, is characterized by decreased or absent sperm motility in the ejaculate [3]. Sperm motility is a prerequisite that ensures the migration of spermatozoa from the vagina to the Fallopian tubes, penetration into the cumulus oophorus, and completion of fertilization. The relationship between impaired sperm motility and reduced probability of natural conception has been well established [4]. Asthenozoospermia is a complex disorder with a multifactorial and complex aetiology that can be classified into intrinsic and extrinsic factors. Intrinsic factors encompass a spectrum of disorders, including tail anatomical abnormalities [5, 6], antisperm antibodies [7], mitochondrial dysfunction [8], Kartagener’s syndrome [9], and a heterozygous variant of DnaJ heat shock protein family member B13 [10]. On the other hand, extrinsic factors mainly consist of genital infections [11], cigarette smoking [12], pesticide exposure [13], and environmental pollution [14].

First introduced by Palermo et al. [15] in 1992, intracytoplasmic sperm injection (ICSI) has become an invaluable technique for couples with severely compromised semen parameters to produce biological offspring. The first reported clinical pregnancy of ICSI using testicular spermatozoa in couples with complete asthenozoospermia was addressed in 1996 [16]. Since then, many studies have demonstrated that testicular spermatozoa offer a better ICSI outcome than ejaculated spermatozoa in couples with complete asthenozoospermia [16–18]. Moreover, some studies suggested comparable outcomes of ICSI when testicular spermatozoa were used regardless of their motility [18]. However, a recent case-control study reported that ICSI outcomes using testicular spermatozoa were comparable to those from ejaculated spermatozoa in men with severe or complete asthenozoospermia [19].

Overall, the rarity of severe or complete asthenozoospermia has hindered our understanding of this clinical entity in the context of assisted reproduction [19, 20]. Whether testicular or ejaculated spermatozoa should be used for a better ICSI outcome in couples with complete asthenozoospermia remain questionable. Moreover, there are insufficient data for determining whether different extents of severe or complete asthenozoospermia could affect the ICSI outcomes. Therefore, the purpose of this study was to compare ICSI outcomes from the use of testicular spermatozoa with those from ejaculated spermatozoa in men with complete asthenozoospermia and explore whether different extents of severe or complete asthenozoospermia could affect the ICSI outcomes. We present the following article in accordance with the STROBE reporting checklist.

## Materials and methods

### Study patients

This study enrolled male patients with severe asthenozoospermia (0 < the percentage of progressively motile spermatozoa ≤ 1%) [19, 21] or complete asthenozoospermia who were admitted to our center between January 2014 and December 2018 and underwent ICSI during this period. Medical records, including baseline characteristics, hormone profile, semen parameters, normal fertilization, good-quality embryos on day 3, transferred embryos, and ICSI outcomes were retrospectively evaluated. On the day of oocyte retrieval, the male patient was asked to provide a second semen sample if only immotile spermatozoa were detected in the first semen sample. If there were only immotile spermatozoa in both the semen samples, the couple was given four options: ICSI using ejaculated spermatozoa, ICSI using testicular spermatozoa, oocyte freezing, and in-vitro fertilization (IVF) using donor spermatozoa. SperMagic medium containing sperm stimulators and nutritional elements was selected for treating immotile spermatozoa. This medium has been widely reported for human ICSI [22–24] and does not increase the incidence of adverse ICSI outcomes [24]. In our center, only motile spermatozoa with/without treatment with SperMagic medium were used for ICSI. In other words, if there were no motile testicular/ejaculated spermatozoa even after treatment with SperMagic medium, the couple would have to select oocyte freezing or IVF using donor semen.

### Inclusion criteria

According to the sperm category used in ICSI, patients with severe or complete asthenozoospermia were further classified into four groups: ejaculated progressive motile sperm group (Ep-group), ejaculated non-progressive motile sperm group (En-group), ejaculated immotile sperm group (Ei-group), and testicular sperm group (TESE-group). Specifically, patients in the Ep-group included those who underwent ICSI using ejaculated progressive motile spermatozoa, whereas patients in the En-group included those who underwent ICSI using ejaculated non-progressive motile spermatozoa. Besides, patients were included in the Ei-group when all the three items in the following criteria were concurrently met: (1) 100% spermatozoa in both semen samples were immotile before treatment with SperMagic medium; (2) the ejaculated spermatozoa were activated and motile after treatment with SperMagic medium; and (3) ICSI using ejaculated spermatozoa was performed during this period. Furthermore, patients in the TESE-group included those who had testicular spermatozoa with/without treatment with SperMagic medium and underwent ICSI using testicular spermatozoa during this period. Testicular spermatozoa were collected through testicular sperm extraction (TESE), which was performed as described previously [25].

### Semen analysis

Semen analysis was performed in the laboratory department of our center according to the fifth edition of World Health Organization (WHO) guidelines [3]. Specifically, the normal range of spermatozoa parameters were as follows: semen volume, ≥ 1.5 mL; sperm concentration, ≥ 15 million/mL; total sperm motility, ≥ 40%; normal morphology of spermatozoa, ≥ 4% [3]. The number, motility and morphology of spermatozoa were assessed through a computer-aided spermatozoa analysis system (Sperm Class Analyzer, MICROPTIC, Barcelona, Spain). The intra- and inter-assay coefficients of variation were both set to < 15%.

### Hormone profile

After overnight fasting, patients were made to sit for half an hour prior to sampling. Subsequently, blood samples were obtained between 8:00 and 10:00 A.M. Serum levels of follicle-stimulating hormone (FSH), luteinizing hormone (LH), prolactin and total testosterone were determined by the Roche 601 (Roche Diagnostics, Mannheim, Germany) based on electrochemiluminescence assays. Furthermore, the normal range of each hormone was as follows: FSH, 1.5–12.4 IU/L; LH, 1.7–8.6 IU/L; prolactin, 4.04–15.2 ng/mL; total testosterone, 249–836 ng/dL. A second blood sample was collected for re-assessment if abnormal levels of any of these hormones were detected.

### ICSI outcomes

Details pertaining retrieved oocytes, MII oocytes, normal fertilization, fertilized oocyte, good-quality embryos on day 3, transferred embryos, biochemical pregnancy, clinical pregnancy, ongoing pregnancy, and live birth were recorded for assessing ICSI outcomes. Biochemical pregnancy was established when serum human chorionic gonadotropin level was over 10 mIU/mL. Clinical pregnancy was confirmed when the gestational sac was observed in an ultrasound examination. Ongoing pregnancy was determined by identification the fetal heartbeat on ultrasound at 12 weeks of gestation. Live birth was established when a live-born infant(s) was delivered after 28 weeks of gestation or more.

### Testicular volume and body mass index (BMI)

The testicular volume was assessed by scrotal ultrasonography and determined by the length × width × depth × 0.71 [26]. The normal range of testicular volume was 15–25 mL [27]. In addition, BMI was computed as body weight in kilograms divided by body height in meters squared (kg/m^2^). BMI in adults was defined as < 18.5 for underweight, 18.5–24.9 for normal, 25.0–29.9 for overweight, and ≥ 30.0 for obesity [28].

### Statistical analysis

Data analysis was performed with SPSS (version 22.0, Chicago, USA). Continuous variables were described as means ± SD, and Mann–Whitney rank sum test or Kruskal–Wallis test was used to compare the data of different groups. Categorical variables were described as the frequency with a percentage, and between-group difference was evaluated with χ^2^ test or Fisher’s exact test.

## Results

### Baseline characteristics and semen parameters

A total of 97 couples with severe or complete asthenozoospermia were enrolled in this study. Of the 79 couples who underwent ICSI using ejaculated spermatozoa, progressively motile spermatozoa were used in 25, whereas non-progressively motile spermatozoa and immotile spermatozoa were used in 43 and 11, respectively. Moreover, 18 couples underwent ICSI using testicular spermatozoa. Table 1 summarizes the baseline characteristics of the men with severe or complete asthenozoospermia. The semen parameters of the patients are also listed in Table 2. No significantly between-group difference was observed.

**Table 1:** Baseline characteristics of couples with severe or complete asthenozoospermia

Characteristics	Ejaculated spermatozoa			TESE	*P* value^*^
	Ep	En	Ei		
Number of couples	25	43	11	18	-
Age of husband (years) mean ± SD	32.16 ± 5.40	30.14 ± 4.28	32.27 ± 3.00	30.56 ± 3.49	0.213
Age of wife (years) mean ± SD	29.48 ± 3.50	29.05 ± 4.40	30.09 ± 3.24	30.33 ± 4.16	0.671
Past pregnancy of wife, n (%)					
Primary	19 (76.00)	35 (81.40)	9 (81.80)	15 (83.30)	0.945
Secondary	6 (24.00)	8 (18.60)	2 (18.20)	3 (16.70)	
BMI of husband (kg/m^2^) mean ± SD	24.03 ± 4.23	25.83 ± 3.68	23.26 ± 4.00	26.41 ± 3.98	0.057
Testicular volume (mL), mean ± SD					
Left	15.36 ± 2.87	16.09 ± 3.48	14.91 ± 3.89	16.61 ± 3.65	0.485
Right	15.44 ± 3.72	16.33 ± 3.53	14.73 ± 4.27	16.51 ± 3.69	0.443
Sexual hormone of husband, mean ± SD					
FSH (IU/L)	5.49 ± 2.48	5.51 ± 4.79	6.18 ± 3.74	5.14 ± 2.92	0.926
LH (IU/L)	5.43 ± 2.34	6.75 ± 5.79	6.97 ± 2.85	5.85 ± 3.68	0.623
Total testosterone (ng/dL)	478.27 ± 230.43	527.09 ± 281.67	428.27 ± 307.41	517.77 ± 279.20	0.719
Prolactin (ng/mL)	10.74 ± 4.43	12.47 ± 6.12	11.42 ± 5.79	10.56 ± 4.49	0.603

*BMI* body mass index, *Ei* ejaculated immotile spermatozoa, *En* ejaculated non-progressively motile spermatozoa, *Ep* ejaculated progressively motile spermatozoa, *FSH* follicle-stimulating hormone, *LH* luteinizing hormone, *SD* standard deviation, *TESE* testicular spermatozoa

^*^There were two types of *P* values, one for the Kruskal–Wallis test for continuous variables and another for the Fisher’s exact test for categorical variables

**Table 2:** Semen parameters of patients with severe or complete asthenozoospermia

Contents	Ejaculated spermatozoa			TESE	*P* value^*^
	Ep	En	Ei		
Number of patients	25	43	11	18	-
Semen volume (mL) mean ± SD	2.18 ± 0.98	2.53 ± 1.44	2.80 ± 1.45	2.44 ± 1.59	0.407
Sperm concentration (million/mL) mean ± SD	17.57 ± 15.32	13.22 ± 12.41	18.75 ± 14.91	15.33 ± 13.67	0.348
Sperm motility (%)^†^					
PR	0.83 ± 0.27	0	0	0	-
NP	1.82 ± 1.45	1.73 ± 1.12	0	0	-
IM	97.35 ± 1.30	98.27 ± 1.12	100	100	-
Abnormal morphology (%) mean ± SD	88.67 ± 1.21	90.92 ± 4.84	93.00 ± 4.24	91.78 ± 4.64	0.314
HOST (%)	64.88 ± 18.18	65.86 ± 16.65	59.18 ± 20.35	58.83 ± 17.68	0.423

*Ei* ejaculated immotile spermatozoa, *En* ejaculated non-progressively motile spermatozoa, *Ep* ejaculated progressively motile spermatozoa, *HOST* hypo-osmotic swelling test, *IM* immotile, *NP* non-progressively motile, *PR* progressively motile, *SD* standard deviation, *TESE* testicular spermatozoa

^*^Kruskal–Wallis test was performed to detect the between-group difference. ^†^The sperm motility of testicular spermatozoa was based on that before treatment with the SperMagic medium

### Fertilization, embryos and ICSI outcomes

Among the couples enrolled, there were 113 cycles in total. Data pertaining to normal fertilization, good-quality embryos on day 3, and transferred embryos are summarized in Table 3. In addition, ICSI outcomes of the patients with severe or complete asthenozoospermia are listed in Table 4. The clinical pregnancy rate was significantly higher in the Ep-group (65.4%, *P* = 0.019) and the TESE-group (63.6%, *P* = 0.035) than in the Ei-group (23.1%). In addition, the ongoing pregnancy rate in the Ei-group was significantly lower than that in the Ep-group (23.1% vs. 61.5%, *P* = 0.041). In the Ep group, pregnancy loss was observed in three cycles (11.5%), and live birth was achieved in 15 cycles among which 3 gave birth to twins. In the En-group, 10 cycles (19.2%) resulted in pregnancy loss, and live birth was achieved in 21 cycles among which 2 gave birth to twins. One neonate of the twins was diagnosed with chondrodysplasia, and another singleton was born with patent foramen ovale. In the Ei-group, one of the three singletons was diagnosed with right cryptorchidism. Moreover, the TESE group had 10 singletons of whom one was diagnosed with anal atresia.

**Table 3:** Normal fertilization, good-quality embryos on day 3 and the transferred embryos

Contents	Ejaculated spermatozoa			TESE	*P* value^*^
	Ep	En	Ei		
Number of cycles	26	52	13	22	-
Number of retrieved oocytes mean ± SD	11.12 ± 5.26	10.40 ± 5.73	11.08 ± 6.78	10.65 ± 4.32	0.957
Number of MII oocytes mean ± SD	9.44 ± 4.62	8.92 ± 5.14	7.00 ± 3.94	8.24 ± 4.15	0.483
Normal fertilization rate (%)^†^	157/236 (66.5)	272/428 (63.6)	54/91 (59.3)	112/173 (64.7)	0.638
Number of fertilized oocytes mean ± SD	6.04 ± 3.47	5.91 ± 4.16	4.15 ± 2.30	4.33 ± 3.57	0.254
Rate of good-quality embryos on day 3 (%)^‡^	93/157 (59.2)	150/272 (55.2)	25/54 (46.3)	70/112 (62.5)	0.204
Number of good-quality embryos on day 3 mean ± SD	3.58 ± 2.45	3.19 ± 2.83	1.92 ± 1.59	3.68 ± 2.96	0.278
Number of transferred embryos mean ± SD	1.50 ± 0.51	1.49 ± 0.78	1.23 ± 0.73	1.14 ± 0.89	0.248

*Ei* ejaculated immotile sperm, *En* ejaculated non-progressively motile spermatozoa, *Ep* ejaculated progressively motile spermatozoa, *SD* standard deviation, *TESE* testicular spermatozoa

^*^Kruskal–Wallis test was performed to detect the between-group difference. ^†^Normal fertilization rate was computed as the total number of normal fertilized oocytes divided by the total number of MII oocytes. ^‡^The rate of good-quality embryos on day 3 referred to the total number of good quality embryos on day 3 divided by the total number of all embryos on day 3

**Table 4:** ICSI outcomes of patients with severe or complete asthenozoospermia

Outcome	Ejaculated spermatozoa			TESE	*P* value^*^
	Ep	En	Ei		
Number of cycles	26	52	13	22	-
Embryo transfer cycles, n (%)					
Fresh embryo transfer cycles	18 (69.2)	33 (63.5)	8 (61.5)	13 (59.1)	0.902
Frozen embryo transfer cycles	8 (30.8)	19 (36.5)	5 (38.5)	9 (40.9)	
Outcomes, n (%)					
Biochemical pregnancy	18 (69.2)	31 (59.6)	4 (30.8)	14 (63.6)	0.140
Clinical pregnancy	17 (65.4)	24 (46.2)	3 (23.1)^a^	14 (63.6)^b^	**0.046**
Ongoing pregnancy	16 (61.6)	21 (40.4)	3 (23.1)^a^	11 (50.0)	0.116
Live birth	15 (57.7)	21 (40.4)	3 (23.1)	11 (50.0)	0.183
Birth weight of singleton (kg) mean ± SD	3.40 ± 0.52	3.30 ± 0.51	3.39 ± 0.27	3.38 ± 0.49	0.956

*Ei* ejaculated immotile spermatozo, *En* ejaculated non-progressively motile spermatozoa, *Ep* ejaculated progressively motile spermatozoa, *SD* standard deviation, *TESE* testicular spermatozoa. ^a^Significant difference when compared with data from the Ep-group. ^b^No significant difference when compared with the data from the Ei-group

^*^Mann–Whitney rank sum test was performed to detect the difference between the two groups, while Kruskal–Wallis test was performed to detect the difference among the four groups

## Discussion

Asthenozoospermia is a frequently encountered disorder and can be diagnosed in more than 40% of infertile men [29]. On the basis of the extent of impairment of sperm motility, asthenozoospermia is graded as mild, moderate, severe, and complete [19, 27, 30]. For natural conception, rapid-progressively motile spermatozoa are critical for the penetration of cervical mucus [31]. As less than 1% of the ejaculated spermatozoa are progressively motile in men with severe asthenozoospermia, ICSI is a suitable choice for them. The overall semen quality does not affect the ICSI outcome, and live birth can be achieved through ICSI in men with severe asthenozoospermia [32]. However, whether couples with different extents of severe asthenozoospermia have comparable ICSI outcomes remains to be determined. Our data demonstrated that the outcome of progressive ejaculated spermatozoa-ICSI was comparable to that of non-progressive ejaculated spermatozoa-ICSI, and the ICSI outcome was therefore not affected by the extent of severe asthenozoospermia. Sperm vitality is essential to achieve a satisfactory outcome of ICSI. At our center, in order to ensure vitality of the spermatozoa, only motile spermatozoa with/without treatment with SperMagic medium were used for ICSI.

Although the detailed mechanisms of complete asthenozoospermia have not been fully elucidated, a past study has demonstrated that the leading causes of complete asthenozoospermia are ultrastructural abnormalities in the sperm flagellum and necrozoospermia [33]. Ultrastructural defects generally result from spermiogenetic dysfunction [17], wherein the ejaculated spermatozoa are viable but immotile. It should be noted that Kahraman et al. [16] divided the immotile spermatozoa into “initially immotile spermatozoa” (initially immotile but showed some motility after stimulation) and “totally immotile spermatozoa” (immotile before and after stimulation). Nijs et al. [17] argued that the ICSI outcome was much better after using “initially immotile spermatozoa” than “totally immotile spermatozoa”. In our center, immotile spermatozoa after treated with SperMagic medium was not used in ICSI, and ejaculated spermatozoa used in the Ei-group may be considered “initially immotile spermatozoa”. Our data demonstrated that the Ei-group had a significantly lower clinical pregnancy rate compared with the TESE. Moreover, the biochemical pregnancy, ongoing pregnancy, and live birth rates were lower in the Ei-group than in the TESE-group, although these differences were not statistically significant. This is probably caused by a lack of statistical power that necessitates a larger sample size. Collectively, our results suggested that a better ICSI outcome can be achieved using testicular spermatozoa compared with ejaculated spermatozoa in men with complete asthenozoospermia, which is similar to that observed in previous studies [16–18]. It was hypothesized that the vitality of testicular spermatozoa is much higher compared to ejaculated sperm in patients with complete asthenozoospermia [34]. Immotile spermatozoa in the ejaculate might have poor vitality even after treatment with SperMagic medium. On the other hand, immotile testicular spermatozoa are immature but have potential to gain motility after maturation or stimulation. Ortega et al. [33] reported that TESE is also suitable for patients with normal testicular function as long as ejaculated spermatozoa are scarcely viable. In these cases, TESE often yields more viable spermatozoa. Despite these observations, Al-Malki et al. recently reported that in men with severe or complete asthenozoospermia, the outcomes were comparable between testicular spermatozoa-ICSI and ejaculated spermatozoa-ICSI [19]. One possible reason for the different results was that they did not further divide these couples into severe and complete asthenozoospermia groups, and progressively ejaculated spermatozoa-ICSI, non-progressively ejaculated spermatozoa-ICSI, and immotile ejaculated spermatozoa-ICSI were clubbed into one group [19].

The present study has some limitations. First, DNA fragmentation of spermatozoa was not determined in these patients. In addition, stage of the embryo, abortion rate, and implantation rate were not taken into consideration in this study. Furthermore, the sample size of each group in this study is relatively small because the presentation of severe or asthenozoospermia is rare [19, 20], which can be overcome by future multi-center studies.

## Conclusions

Testicular spermatozoa should be preferred to ejaculated spermatozoa for obtaining a better ICSI outcome in couples with complete asthenozoospermia. The ICSI outcomes using testicular spermatozoa in couples with complete asthenozoospermia are comparable to those using ejaculated spermatozoa in couples with severe asthenozoospermia.

## Data Availability

All data are available from the authors on reasonable request.

## Abbreviations

BMI: Body mass index
Ei-group: ejaculated immotile sperm group
En-group: ejaculated non-progressive motile sperm group
Ep-group: ejaculated progressive motile sperm group
FSH: follicle-stimulating hormone
HOST: hypo-osmotic swelling test
ICSI: intracytoplasmic sperm injection
IM: immotile
IVF: in-vitro fertilization
LH: luteinizing hormone
NP: non-progressively motile
PR: progressively motile
TESE: testicular sperm extraction
TESE-group: testicular sperm group
WHO: World Health Organization
